# Harnessing marine-derived materials for therapeutics innovations: Advances in biomaterials from the ocean

**DOI:** 10.1016/j.mtbio.2025.102375

**Published:** 2025-10-09

**Authors:** Mohammad Saeed, Shabnam Anjum, Yang Zhang

**Affiliations:** a School of Dentistry, Shenzhen University Medical School, Shenzhen, 518055, China; b School of Biomedical Engineering, Shenzhen University Medical School, Shenzhen, 518055, China; c Institute of Oral Science, Shenzhen University, Shenzhen, 518055, China; d Department of Stomatology, Shenzhen University General Hospital, Shenzhen University, Shenzhen, 518055, China

**Keywords:** Marine biomaterials, Bioactive scaffolds, Tissue engineering, Drug delivery, Wound healing

## Abstract

Marine-derived biomaterials have gained increasing attention in recent years because of their distinct biochemical composition, biological functionality, and ecological sustainability. Extracted from diverse marine organisms such as algae, crustaceans, mollusks, and fish, these materials particularly polysaccharides, proteins, minerals, and lipids, exhibit favorable properties including biocompatibility, biodegradability, low immunogenicity, and intrinsic bioactivity. Their structural and functional diversity enables their use in a wide range of biomedical applications, including tissue engineering, drug delivery, wound healing, and regenerative medicine. This review provides a comprehensive overview of the current state of research on marine biomaterials, emphasizing their physicochemical properties, mechanisms of action, and biomedical potential. Particular attention is given to recent innovations, emerging challenges, and strategies for future development in the field of biomaterials. The integration of marine-derived components into biomedical systems not only enhances material performance but also offers new pathways for creating sustainable and clinically effective therapies. Through this analysis, we aim to highlight the promise of marine biomaterials as next-generation platforms in biomedical applications.

## Graphical abstract

**Figure:**
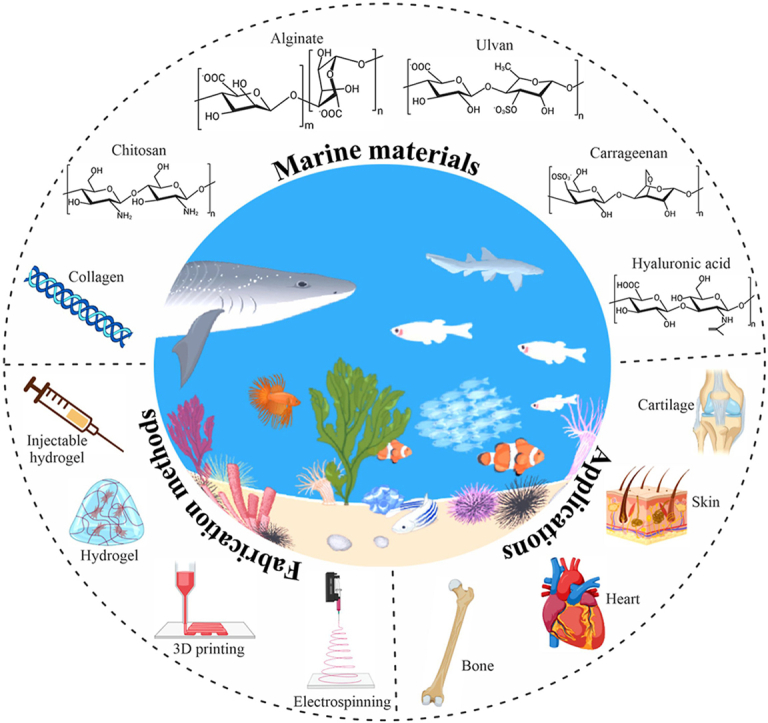

## 1 Introduction

The ocean, which is the planet’s largest ecosystem, is rich in biodiversity and harbors an immense range of biological species, accounting for approximately two-thirds of global biodiversity. It is estimated that there are 2.2 million different kinds of species in the marine environment; however, 91 % of them have yet to be described [1,2]. The marine environment is a unique reservoir of bioactive substances with enormous potential for use in biomedicine. Marine organisms have produced a wide range of physically and functionally distinct biomaterials through the evolution of unique biochemical adaptations. Marine ecosystems have yielded the identification of over 25,000 biologically active compounds. The first marine-derived biomaterials (such as holothurin, the first biologically active substance extracted from a marine origin) were used as anti-tumor agents in 1967 in vivo cancer models.

**Fig. 1:**
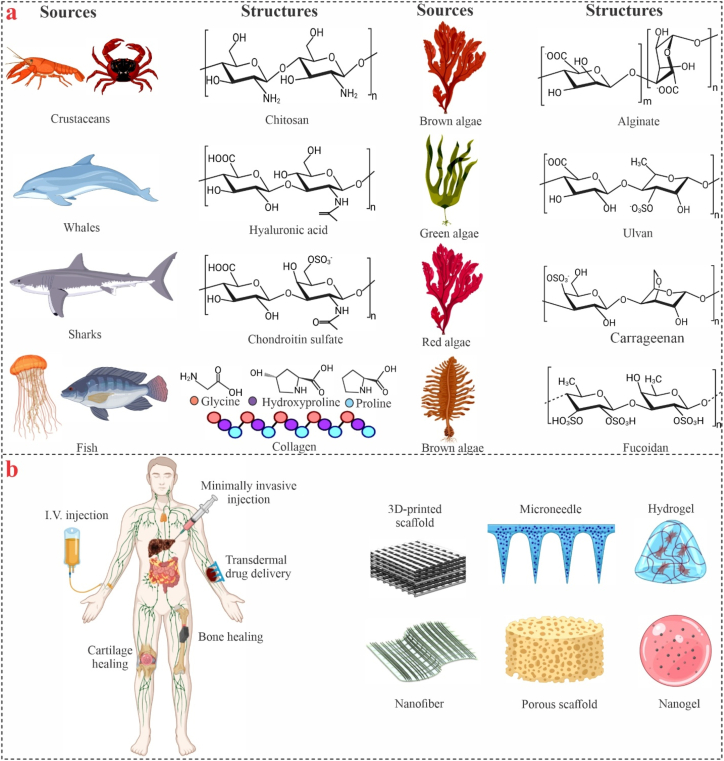
Summary of the most commonly used biomaterials from marine sources and their applications.

Biomaterials derived from marine sources have a lower risk of spreading disease. The development and applications of these biomaterials have been rapidly explored since then. These naturally occurring materials exhibit remarkable biocompatibility, bioactivity, and tunable physicochemical properties, making them highly promising candidates, akin to tissue engineering, controlled drug delivery, wound healing, dental procedures, and orthopedic treatments (Table 1) [[3], [4], [5], [6]]. Researchers have made significant efforts to design and construct sophisticated engineered nanomaterials, aiming to enhance their physicochemical properties to create drug carriers, microspheres, microneedles, hydrogels, nanoparticles, and scaffolds that are suited for individualized and targeted treatments (Fig. 1) [[7], [8], [9]].

**Table 1:** The most recent developments in marine-based biomaterials for biomedical applications.

Marine material	Processing methods	Application	Characterization methods	Results	Ref.
Alginate	Hydrogel	Bone and cartilage	SEM, swelling ratio, rheological characterization, osteogenic, and chondrogenic differentiation, RT-qPCR	Enhanced secretion of osteogenic/chondrogenic ECM promotes differentiation-specific gene expression and facilitates nuclear localization of YAP and TAZ.	[10]
Alginate	Hydrogel	Intervertebral disc degeneration	TEM, SEM, XRD, XPS, rheological analysis, antioxidant assay, RT-qPCR, western blot analysis	Hydrogels proficiently ensnare senescent cells owing to their surface roughness.	[11]
Alginate	Hydrogel	Endothelial cell functionality	Mechanical, physical, optical properties, western blot, RT-qPCR	Maintain cell phenotype, high cell density, and have the possibility of suppression of EnMT-related signaling factors in hCECs.	[12]
Chitosan	Electrospinning	Skin tissue engineering	SEM, FTIR, ^1^HNMR, XPS, porosity, mechanical, swelling, and cytocompatibility test	Superior mechanical performance supports cell viability, proliferation, and differentiation.	[13]
Fish gelatin	3D printing	Tissue engineering	Rheological characterization, mechanical, swelling, and cytotoxicity properties	Excellent cytocompatibility enables their use in 3D cell entrapment and long-term culturing.	[14]
Fish gelatin	Freeze-drying	Skin	SEM, FTIR, mechanical test, swelling, porosity, proliferation, and infiltration analysis	Exhibited good mechanical properties without contraction, and might be used as a basic material for 3D skin models.	[15]
Coral protein	Scaffold	Bone tissue engineering	Transcriptome sequencing, mass spectrometry, molecular docking, micro-CT, and histological analysis	Enhanced the mitochondrial function of pre-osteoblasts and protected them from oxidative stress.	[16]

This review explores the current advancements in the use of marine-derived biomaterials within the biomedical field, with a focus on their structural complexity, functional diversity, and broad applicability. By exploring their structural characteristics, biological properties, and emerging applications, we highlight the potential of marine-derived biomaterials as versatile and sustainable platforms for next-generation biomedical therapies. We emphasized numerous relevant marine biomaterials-related examples to show the potential of these powerful bio-derived materials. We anticipate that marine biology researchers will find the review interesting, and it will offer theoretical direction for the creation of sophisticated marine biomaterials and their eventual clinical use for public health.

## 2 Polysaccharides as biomaterials from marine organisms

### 2.1 Chitin and chitosan

Chitin is the second most abundant natural polysaccharide on Earth, after cellulose [17]. It is primarily derived from the exoskeletons of marine crustaceans such as crabs and shrimps, and is also found in the cell walls of fungi and certain species of green algae. Chitin is a semi-crystalline homopolymer composed of β-(1 → 4)-linked N-acetyl-D-glucosamine units [18]. Chitosan is a naturally occurring amino-polysaccharide consisting of (1 → 4)-linked 2-acetamido-2-deoxy-D-glucosamine units, typically produced by the deacetylation of chitin. In nature, chitin and chitosan are found in various structural forms, including pure chitin, nano-organized chitin-protein complexes, chitin–pigment conjugates, and chitin–mineral composites. Among these, pure chitin demonstrates the highest resistance to alkaline conditions. The molecular weight and degree of deacetylation significantly influence the physicochemical and biological characteristics of both chitin and chitosan, affecting parameters such as solubility, hydrophilicity, and cellular interactions. Chitin generally possesses higher mechanical strength due to its rigid crystalline structure, but chitosan’s mechanical behaviors can be adjusted through deacetylation. Chitin-based scaffolds play an important role in calcium biomineralization and biosilica production. Marine-derived chitin has low cytotoxicity, immunogenicity, potential to promote cell growth and attachment, and degrades naturally within the human body, avoiding the need for surgical removal [19].

Compared to its precursor chitin, marine-derived chitosan demonstrates superior antibacterial efficacy, primarily attributed to the presence of positively charged amino groups that facilitate interactions with negatively charged microbial cell membranes [20]. Furthermore, because chitosan has an amine group of its own, it can undergo a Schiff base reaction with other polysaccharides that have aldehyde groups, making it a useful ingredient for creating self-healing hydrogels. Chitosan’s positive charge can react with the negatively charged phospholipid membrane of extracellular vesicles (EVs), which holds great promise for application in EVs distribution [21]. Shen et al. [22] treated periodontitis with chitosan hydrogel embedded with dental pulp stem cells-derived exosomes (DPSC-Exos) and demonstrated that DPSC-Exos influenced macrophage phenotypes, as well as hydrogels sped up the recovery of alveolar bone and periodontal epithelium in periodontitis-affected mice while preventing the growth of periodontitis.

**Fig. 2:**
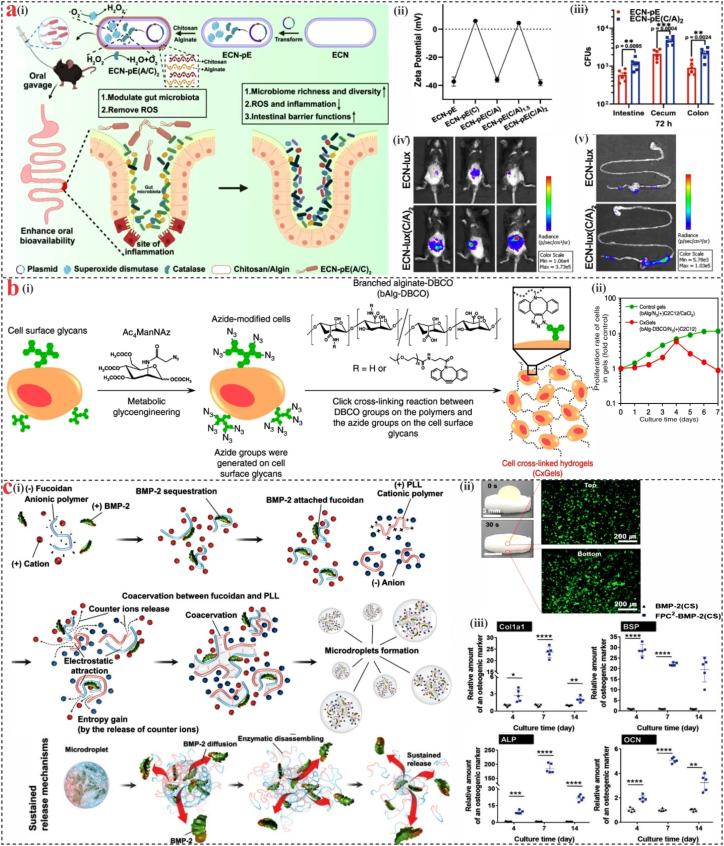
**Marine polysaccharide biomaterials.** (a) (i) Layer-by-layer encapsulation of engineered probiotics utilizing SA and chitosan, (ii) Zeta potential of ECN-pE through various coatings of chitosan/SA layers, (iii) Quantification of live ECN-pE in the stomach, intestine, colon, and cecum 72 h after oral gavage, (iv) IVIS bioluminescence images of mice and their (v) GI tract 3 h after oral gavage. Reproduced with permission [23]. **(b)** (i) Graphical representation of the cell cross-linked hydrogel (CxGels) process, (ii) Proliferation rates of C2C12 cells existing in the CxGels analyzed using the WST-1 assay [36]. **(c)** (i) The sustained protein process and BMP-2 encapsulation development in the fucoidan/poly-L-lysine (PLL) complex coacervate system, (ii) Fluorescence imaging of FPC2-BSA-coated collagen sponge after 1 h of incubation [37].

However, potential applications of chitin and chitosan are hindered by their limited solubility. Significantly extensive endeavors have been undertaken to augment their water solubility and antimicrobial attributes. One approach involves functionalizing chitosan by incorporating cationic and anionic groups. The addition of cationic groups, including quaternary ammonium functionalities, and the introduction of metal/metal oxide nanoparticles, have seemingly heightened the antibacterial effectiveness of chitosan. An alternative strategy to improve the water solubility of chitosan involves the reduction of the positive charges associated with its protonated amino groups on the polymer surface. Chitosan has been modified with sulfonate groups to create anionic chitosan, which has better water solubility and selective antibacterial action. Zhou et al. [23], genetically engineered *Escherichia coli* Nissle 1917 (ECN) to overexpress catalase and superoxide dismutase, generating a recombinant strain (ECN-pE) for treating intestinal inflammation. The layer-by-layer electrostatic self-assembly approach with sodium alginate (SA) and chitosan as coating materials improves the stability and bioavailability of ECN-pE within the gastrointestinal (GI) tract (Fig. 2a). These findings pave the way for advanced oral probiotic delivery systems with enhanced stability and targeted action, offering valuable insights for future clinical applications in GI health and inflammatory disorders.

### 2.2 Glycosaminoglycans

Glycosaminoglycans (GAGs) are linear, negatively charged polysaccharides that are abundant in the extracellular matrix (ECM) and on the cell surfaces of animals, which have a major effect on pathogen-host interactions and cellular mechanisms. The fundamental structure of GAGs consists of a repetitive arrangement of disaccharide units, each comprising an amino sugar and either uronic acid or galactose [24]. GAGs alone or in blends with other biomaterials are increasingly known for their essential roles in regulating cellular communication, differentiation, and proliferation. The main GAGs type includes hyaluronic acid (HA), chondroitin sulfate (CS), heparin and heparan sulfate (HS), dermatan sulfate (DS), and keratan sulfate (KS), each contributing uniquely to the structural and functional dynamics of the ECM [25]. Numerous cellular regulatory processes, such as signaling, cytoskeletal dynamics, receptor activation, and cellular communication, depend on these complexes. Specifically, HA, HS, and CS have been the most widely explored for growth factor delivery applications and incorporated into hydrogels or other biomaterial formulations. KS, a sulfated GAGs, is present in marine sources such as shark cartilage, zebrafish, and the skin of various teleost fish. However, to date, the therapeutic potential of marine-derived KS remains largely unexplored in biomedical research. The GAGs include CS derived from shark cartilage, HS derived from scallops and shrimp heads, and DS from shark skin. CS consists of disaccharide units with sulfate groups at various positions, such as N-acetyl-β-d-galactosamine and β-d-glucuronic acid residues [26]. CS made from marine species (usually extracted and refined from cartilaginous fishes, especially sharks) contains a higher proportion of disulfate units than CS collected from terrestrial sources, which comprises non-sulfated or monosulfated units. In order to manage osteoarthritis, CS was created as an injectable hydrogel that helps maintain its chondrogenic nature. A hydrogel system for encapsulating liquiritin (LQ) loaded liposomes was created by covalently modifying CS with photo-cross-linkable methacryloyl groups (ChsMA) using alginate as a sacrificial template. The persistent release of the encapsulated therapeutic drug is facilitated in an inflamed articular cavity by hyaluronidase’s enzymatic degradation of the CS component, which specifically targets the β-N-acetylhexosamine-1,4 glycosidic linkages. Osteoarthritis is slowed down by the LQ medication’s synergistic elimination of ROS through the breakdown products of CS [27].

Marine-derived HA is sourced from seaweed, fish eyes, whales, shellfish, sharks (skin and cartilage), swordfish, zebrafish, mollusk bivalve, and the liver of stingrays, consisting of long chains of polysaccharides. HA, also referred to as hyaluronan, is a class of non-sulfated linear polysaccharides made up of N-acetylglucosamine (GlcNAc) and glucuronic acid [28,29]. HA, a major component in skin, joints, and eyes, has special viscoelastic qualities that serve to minimize shear stress. HA contributes to the elasto-viscous characteristics of connective tissues and is essential for mediating water transport and preserving tissue hydration. Furthermore, it facilitates the supramolecular arrangement of proteoglycans in the ECM. Significantly, HA has concentration-dependent antibacterial, antibiofilm, and anti-adhesive properties against *Staphylococcus aureus*, showing its potential to prevent wound infections [30]. Strong antibiofilm and antifouling properties are produced by the electrostatic repulsion of HA’s negative charges with negatively charged bacterial cell walls [31]. In the treatment of osteoarthritis, intra-articular administration of HA is frequently employed to alleviate inflammation and support tissue regeneration [32,33]. For instance, low molecular weight HA has been demonstrated to have a dose-dependent influence on cellular function by increasing osteocalcin mRNA expression and promoting cellular proliferation [34]. Additionally, studies show that HA can be functionally modified with sulfated biomolecules, including CS, perlecan, and heparin, to increase its binding affinity for significant growth factors. This modification not only improves bioactivity for skeletal tissue engineering but also enables a more sustained and controlled release of these growth factors [35].

### 2.3 Alginate

Alginates are linear anionic polysaccharides composed of (1–4)-linked β-D-mannuronic acid and α-L-guluronic acid monomer units [38]. These compounds found in marine brown algae (*phaeophyceae)* and soil bacteria, exhibit valuable traits such as gelling, viscosity adjustment, good porosity, and biocompatibility, which make them appropriate for a range of pharmacological and biological uses. Alginates can retain water up to almost 300 times their molecular weight, a remarkable capability. The interaction of divalent cations like Ca^2+^, Sr^2+^, and Ba^2+^ with the flexible guluronic acid segments in the polymer chain is responsible for this characteristic, as well as their tendency to form hydrogels in the presence of these ions [39]. The stiffness of SA hydrogel increases linearly with the amount of divalent cations. Its internal “egg-box” structure remains consistent, unaffected by variations in chelated cation concentration [40]. Research by Wei et al. [41] used Ca^2+^ to adjust the stiffness of SA hydrogels to promote the paracrine activity of bone marrow-derived mesenchymal stem cells (BMSCs). Additionally, the hydrogel was functionalized with the stem cell homing peptide SKPPGTSS to promote BMSCs recruitment. This engineered hydrogel demonstrated immunomodulatory effects and markedly increased bone healing and neovascularization in a rat model of distal femoral defect. These findings suggest that the SA-based hydrogel represents a minimally invasive and effective approach for accelerating the repair of osteoporotic bone defects. Nagahama et al. [36] developed a living alginate hydrogel through a bio-orthogonal click chemistry approach, employing a crosslinking reaction between azide-modified mammalian cells and alkyne-functionalized alginate. Azide groups were added to the cell surface’s sialic acid residues, and a dibenzocyclooctyne (DBCO) derivative was used to create the strained alkyne-functionalized alginate (Fig. 2 b). The logarithmic proliferation of C2C12 cells within this crosslinked gel system (CxGels) suggests that essential cellular processes and viability were maintained within the hydrogel matrix. Gelation is a key characteristic of alginates; it can be created with different functionalities, including adhesive materials. These hydrogels can be tailored to possess specific physical characteristics that guide the growth and differentiation of 3D cells.

### 2.4 Carrageenan

Carrageenan comprises a group of linear, sulfated polysaccharides that function as anionic polymers and are primarily derived from specific red algae (Rhodophyta-Order Gigartinales), such as *Chondrus crispus*, *Eucheuma cottonii*, *Gigartina* spp., and *Spinosum* spp. Carrageenan’s structural components are repeating units of D-galactose and 3,6-anhydrogalactose. It is typically categorized into three main types-kappa (κ), iota (ι), and lambda (λ)-based on the degree of sulfation present in the polymer chain [42]. The use of carrageenan is restricted by their intrinsic hydrophilicity, low water vapor permeability, and water resistance. In order to increase the range of applications for carrageenan, a lot of work has been done to enhance its characteristics in the form of films through blending and the addition of nanoparticles. Biologically, carrageenan showcases a range of activities, such as anti-inflammatory, antimicrobial, antitumor, antioxidant, anti-hyperlipemic, anticoagulant, and immunomodulatory effects. Owing to its capacity to concentrate coagulation components, κ-Carrageenan also has exceptional qualities as a hemostatic dressing material, facilitating quick hemostasis [43]. A nanoengineered hydrogel system containing κ-carrageenan can entrap nanosilicates by electrostatics. This study found that κ-carrageenan increased biocompatibility and mechanical rigidity. Prior research has demonstrated that κ-carrageenan has strong antibacterial qualities, effectively eliminating *Staphylococcus epidermidis* and *Escherichia coli* within a 3-h incubation period. The prepared hydrogel dramatically increased the attachment and growth of firming keratinocytes while decreasing the wound size (from 31 % to 1.3 % of the wound area), indicating a promising method for wound healing [44]. Tytgat et al. [45] create gelatin hydrogel sheets with κ-carrageenan for adipose tissue engineering. The findings demonstrate that, in terms of their potential for adipose tissue engineering, the gelatin and κ-carrageenan blends perform noticeably better than the one-component hydrogels. Li et al. [46] use κ-carrageenan to overcome the restriction of gelatin for biofabrication at 37 °C without the need for further post-crosslinking. The interfacial bonding of a 3D printed multilayered structure was improved via the electrostatic attraction between κ-carrageenan and gelatin, which are cationic and anionic hydrogels. It is discovered that these two hydrogels with opposite charges have a much stronger interfacial bond than either a bilayered κ-carrageenan or a bilayered gelatin. At 37 °C, the multilayered κ-carrageenan-gelatin bioprint exhibits excellent biocompatibility and strong structural integrity [46]. Liang et al. [47] constructed a composite hydrogel using carrageenan with chitosan and found that increasing concentrations of carrageenan led to a significant upregulation in the expression of cartilage-specific genes. Results showed that carrageenan facilitated ATDC5 cells to undergo chondrogenic differentiation and enhanced cellular responses in the composite hydrogel, including attachment, survival, and proliferation [47].

### 2.5 Ulvan

Ulvan is a unique sulfated polysaccharide derived from green algae and consists of sulfated rhamnose, xylose, sulfate, d-glucuronic acid, and l-iduronic acid [48,49]. Ulvan exhibits structural resemblance to GAGs, primarily owing to the presence of glucuronic acid and sulfate moieties within its polysaccharide chains [50]. Ulvan has proven to be a promising nutraceutical substance with positive physiological effects that support disease prevention and health promotion. It has been associated with anti-inflammatory, anticancer, antibacterial, and antiviral activities, in addition to exhibiting immunomodulatory and lipid-lowering properties [51,52]. Chi et al. [53] investigated the antiviral properties of ulvan and its depolymerized derivatives. The high molecular weight fragment ulvan-F1 (38.5 kDa) and the native ulvan (1068.2 kDa) both showed notable antiviral efficacy, which was explained by their capacity to obstruct viral infection and replication processes, most likely by binding to cellular surface receptors or interacting with viral envelope glycoproteins. Ulvan extracted from *U. pertusa, U. rigida*, and *U. prolifera* activates RAW 264.7 cells, increasing the production of enzymes (like iNOS and COX-2), cytokines (like TNF-α, IL-1β, IL-6, and IL-10), and their byproducts (like NO and PGE2) [54]. These mediators are in charge of triggering immunological reactions. In order to accelerate type II diabetic wound healing, Ren et al. [55] created a multifunctional hydrogel matrix using ulvan dialdehyde, chitosan, dopamine, and antibacterial silver nanoparticles (AgNPs). They encapsulated human umbilical cord mesenchymal stem cells (hUC-MSCs), which are used to promote cell growth, in a lyophilized powder form (UC-DPA-Ag@hUC-MSCs). Additionally, ulvan’s biomimetic ability prevents foreign body reaction, supporting in vivo tissue engineering. Studies on skin tissue engineering in Wistar rat models demonstrate that ulvan stimulates angiogenesis [56]. Don et al. [57] created dissolving ulvan microneedles (UMNs) for the first time using a two-step casting process. After just 2 minutes of post-insertion, the needle height had dropped by 90.3 %, demonstrating that the ulvan microneedles could completely penetrate the porcine skin to the dermis layer and dissolve quickly. According to this study, the quickly dissolving UMNs may be able to deliver the medication into the skin efficiently, making them a promising transdermal drug delivery system [57].

### 2.6 Fucoidan

Fucoidans are sulfated polysaccharides predominantly extracted from brown seaweeds and certain echinoderms, such as sea urchins and sea cucumbers. These compounds typically represent approximately 5–10 % of the dry weight of brown algae, with variations influenced by species type and harvesting season. Structurally, fucoidans consist of α-(1 → 3)-linked L-fucopyranose units or alternating sequences of α-(1 → 3)- and α-(1 → 4)-linked L-fucopyranoses.

Additional monosaccharides that are incorporated into the fucosyl backbone of fucoidan structures include xylose, galactose, and uronic acids. The structural analysis of fucoidan is complex because of its heterogeneous composition, which includes minor sugars, non-carbohydrate components, and variations in sulfation and acetylation patterns that heavily rely on the source organism and extraction technique. Fucoidan exhibits the capacity to interact with a range of macromolecules, particularly proteins. These interactions are largely influenced by fucoidan’s negative charge, molecular weight, and degree of sulfation, which collectively determine its binding affinity [58]. Usually, at a concentration of up to 25 %, fucoidan exhibits a limited intrinsic gelation capacity. On the other hand, fucoidan’s negatively charged sulfate groups can electrostatically interact with the positive charge groups of other polymers, such as chitosan, to produce gels and films [59]. Non-gelling fucoidan could form gel by blending with κ-carrageenan [60]. Fucoidan has been shown as an excellent material for the controlled delivery of proteins due to its ability to interact selectively with specific protein domains through a combination of electrostatic forces, van der Waals interactions, and hydrogen bonding [61]. Jeon et al. [37] used the electrostatic interactions between cationic bone morphogenetic protein-2 (BMP-2), anionic fucoidan, and the cationic polymer PLL to create a fucoidan-based complex coacervate system, and made it possible for sustained released proteins to promote bone repair (Fig. 2c). Interestingly, fucoidan-based hydrogels, even in the absence of encapsulated therapeutic agents, have demonstrated efficacy in treating dermal burns and facilitating wound healing [62]. Marinval et al. [63] created a polyelectrolyte multilayer (PEM) film composed of fucoidan and vascular endothelial growth factor (VEGF) to coat valve scaffolds, aiming to enhance the functionality of heart valve bioprostheses. Results suggest that fucoidan/VEGF-based PEM coatings could be a viable approach to enhancing the biocompatibility and regenerative capacity of valve bioprostheses for clinical applications.

The biological activity of fucoidans is significantly influenced by molecular weight [64]. It has been shown that high molecular weight fucoidan (130 kDa) increases cell viability and promotes the release of nitric oxide and interferon-gamma (IFN-γ). In contrast, low molecular weight fucoidan (30 kDa), despite having a similar chemical composition, demonstrates comparatively reduced bioactivity.

## 3 Proteins and peptides biomaterials from marine organisms

### 3.1 Gelatin

Gelatin is mostly made from the tissues of porcine and bovine species, which raises ethical and religious issues (e.g., Halal, Kosher, etc.) as well as the risk of zoonosis. However, fish gelatin is acceptable in several cultures and religions. Marine gelatin made from cold-water fish has been gaining more and more attention. Marine gelatin supplies include squids, sponges, fish skins, and jellyfish [65]. Fish gelatin contains lower levels of proline and hydroxyproline than mammalian gelatin, which lowers the gelation and melting temperatures [66]. Gelatin from cold-water fish skin does not gel at normal temperatures; instead, it gels at temperatures between 8 and 10 °C. The ability to create fish gelatin-hydrogels at comparatively low temperatures makes them more suitable for loading with therapeutic medicines that are sensitive to heat. In soft tissue engineering, fish gelatin hydrogels are more suited for creating certain tissue analogs because of their high viscosity and poor mechanical qualities. It finds extensive use in clinical platforms, serving as a stabilizing agent in therapeutic plasma expanders.

The rigidity and quick biodegradation of fish gelatin are its drawbacks in tissue engineering. To address these issues, cross-linking techniques can raise the denaturation temperature and improve the stability of the gelatin molecule, making it appropriate for therapeutic use [67]. Maihemuti et al. [68] used cold-water fish skin gelatin to create a 3D printed porous multilayer scaffold for the healing of osteoarticular cartilage. In order to enhance viscosity, printability, and mechanical strength, cold-water fish skin gelatin was combined with SA and a double-crosslinking procedure. These scaffolds replicate the natural cartilage network’s structure in a way that facilitates chondrocyte adhesion, proliferation, and communication, nutrition delivery, and joint preservation [68].

Shen et al. [69] created a multipurpose scaffold produced from fish using decellularized fish scale, methacrylate fish gelatin, and mesenchymal stem cells **(**MSCs). They also added black phosphorus nanosheets to the network. With increased mineralization and osteogenic marker expression, the scaffold produced from fish can aid MSCs in the osteogenesis process in vitro with the use of near-infrared (NIR) light. Additionally, the scaffold with photothermal therapy and MSCs has enhanced the regeneration of animals with calvarial defects [69]. Recent research has shown that peptides obtained from marine creatures such as cobia, cod, tuna, blue mussels, jumbo squid, and others can function as potential antioxidants [[70], [71], [72]]. These peptides are generated from various marine gelatin hydrolysates. By stopping the radical chain reaction of lipid peroxidation, marine gelatin peptides are known to have positive effects on scavenging free radicals and preventing oxidative damage [73]. Gelatin hydrolysate from the skin of Pacific cod (Gadus macrocephalus) has been demonstrated to enhance the transforming growth factor-β/Smad signaling pathway and mitigate inflammatory responses, thereby providing protective effects against UV-induced photodamage in human skin [74]. In conclusion, gelatin derived from marine organisms possesses a high protein content, unique physical properties like higher melting temperatures, variable mineral content, and reduced production costs. Researchers continue to explore its potential, harnessing the unique properties of gelatin to advance various fields. To increase the application potential of marine-derived gelatin, recent gelation investigations have placed a greater emphasis on the production of multicomponent gelatin nanocomposite hydrogels with the goal of producing better tissue substitutes.

### 3.2 Collagen

Collagen, a primary constituent of the ECM, has been extensively utilized as a hemostatic agent in tissue injury due to its vital biological role [75]. Collagen is present across multiple tissues, including bone, skin, tendons, and teeth, and contributes significantly to the body’s structural integrity. Currently, 29 different forms of collagen have been discovered; type I collagen is the most common, especially in the ECM of bone and tendon tissue [76,77]. Marine collagen, which comes from fish, seaweed, sponges, and jellyfish, is far superior to mammalian collagen because it is easily extracted, soluble in water, and has excellent chemical and physical stability. Marine-derived collagen, especially from fish, closely resembles human Type I collagen in terms of its amino acid composition, making it a promising alternative [78]. Additionally, the smaller particle size of marine-derived collagen allows for rapid circulation in the bloodstream and quicker absorption into the human body, surpassing traditional land-based collagen sources. Notably, marine-derived collagen is not only effective but also cost-effective, yielding high quantities [79,80].

Fish collagen has been used for over 70 years, although it is less denatured than its bovine and porcine counterparts. Type I collagen derived from fish offers a promising alternative to traditionally used mammalian collagen, owing to its notable hemostatic properties and effective wound healing capabilities [81,82]. Shi et al. [83] created porous collagen sponge as burn wound dressings using fish scales. The sponge helped a rabbit model with a burn wound heal without leaving any scars. Fish-derived collagen exhibits biocompatibility and immunostimulatory properties on peripheral blood mononuclear cells (PBMCs), facilitating the stimulation and growth of monocytes and lymphocytes, particularly CD8^+^ T cells. It also stimulates cytokine production without exerting cytotoxic effects [84].

Cao et al. [85] developed a controlled release growth factor scaffold by incorporating bFGF-loaded human umbilical vein endothelial cells (HUVEC) microspheres into freeze-dried fish collagen/chitosan/chondroitin sulfate for skin tissue engineering. In vitro and in vivo evaluations showed that the scaffolds were highly biocompatible and promoted fibroblast growth and skin tissue regeneration. Additionally, the peptide chain of the marine collagen hydrolysate has a large number of methionine residues that have been demonstrated to operate as active sites for antioxidant activity [86].

Nevertheless, it has been noted that collagen scaffolds lack mechanical strength, which restricts their use in hard tissue restoration. Cross-linking has been widely employed to enhance mechanical qualities, making it easier to integrate cells and create a stable 3D scaffold. Carvalho et al. [87] involved cryoprocessing marine collagen (jCOL), chitosan (sCHT), fucoidan (aFUC), and chondroitin sulfate (aCS) at −20, 80, and 196 °C. In order to produce cohesive and stable cryogel materials, a crosslinking technique without the use of chemicals was investigated. The cryogels show an intriguing microenvironment that promotes the cells’ vitality and proliferation [87]. Additionally, sturgeon fish collagen fibril hydrogels were investigated as a substitute for cartilage in the body’s load-bearing area. The strength and durability of these hydrogels are comparable to that of actual cartilage, making them load-bearing biomaterials. Results revealed long-lasting, good biomechanical capabilities and an excellent bone attachment capability [88]. However, before this intriguing biomaterial option can be used in regenerative medicine, more proof of the biosafety of collagen obtained from marine sources is required.

### 3.3 Protein-based bioadhesive

**Fig. 3:**
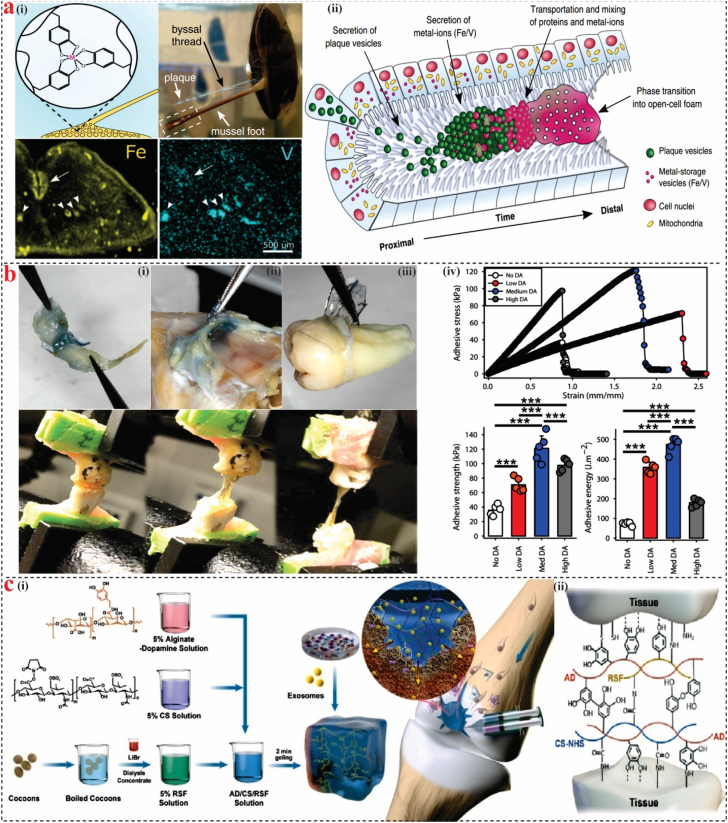
**Marine protein-based bioadhesives.** (**a)** (i) Byssal threads exhibit DOPA-metal coordination cross-linking, a process essential to porous plaque adhesives mechanical strength. The mussel fully extends its foot and uses several byssal strands to anchor itself. The spatial distribution of iron (yellow) and vanadium (turquoise) was determined by elemental analysis of a 200 μm-thick cross-section of the mussel foot, (ii) Metal storage particle secretion was seen inside the longitudinal channels. Reproduced with permission [98]. **(b)** Photographs illustrating hydrogel adhesive properties to (**i**) rat gingival tissue, (ii) at calvarial bone and periosteum, and (iii) the surface of a human tooth root. A tensile test was conducted to evaluate adhesion on rat alveolar bone, (iv) Stress-strain curve was used to quantify adhesion strength and adhesion energy on porcine skin [102]. **(c)** (i) The AD/CS/RSF/EXO hydrogel fabrication method. Reproduced with permission [103]. (For interpretation of the references to color in this figure legend, the reader is referred to the Web version of this article.)

Protein-based bioadhesives have garnered significant attention because of their distinct structural, chemical, and physical attributes, driving advancements in understanding their potential across diverse applications. These adhesives have found particular relevance in the medical field, where the challenges of adhering to wet surfaces within the human body are particularly daunting due to the presence of moisture, which can weaken the adhesive bond. Significant progress in materials science has been achieved through the investigation of the adhesion mechanisms employed by marine mussels to attach to solid substrates [89]. Recently, marine mussels have mastered the art of attaching tenaciously to a variety of surfaces in wet situations [90]. The Mussel adhesion protein (MAP) released by ocean mussels is high in catechol groups [91]. Marine mussels exhibit a remarkable ability to adhere to wet and dynamic underwater surfaces, primarily through the secretion of a particular protein-based holdfast known as the byssus [92]. This structure is anchored to substrates via a complex biochemical mechanism involving a family of mussel foot proteins (Mfps), which are secreted from a specialized organ called the mussel foot [93]. The distal end of the byssus, referred to as the byssal plaque, is uniquely adapted for adhesion and incorporates several distinct Mfps, including mfp-2, mfp-3S, mfp-3F, mfp-4, mfp-5, and mfp-6, particularly in species belonging to the *Mytilus* genus [94,95]. A key feature of these adhesive proteins is their enrichment in the post-translationally modified amino acid *L*-3,4-dihydroxyphenylalanine (DOPA), a catechol-containing residue that is supposed to play a pivotal role in promoting strong interfacial interactions in wet environments [96,97]. Mussels create Byssus fibers, which are mechanically reinforced by protein-metal coordination mediated by DOPA. During the adhesion generation process, metal ions within the microfluidic system are bound by secretory vesicles enriched with concentrated liquid proteins. Protein–metal coordination interactions are facilitated by these ions’ subsequent involvement in the formation of new vesicles (Fig. 3a) [98]. DOPA contributes to adhesion by engaging in various interactions, such as hydrogen bonding, metal coordination, and π-π stacking with a variety of substrates, which collectively enable robust underwater adhesion [99,100]. Ryu et al. [101] developed an intraperitoneal (IP) adhesive patch that adheres strongly to wet tissues via synergistic interactions between chitosan’s amino groups and catechol moieties. The IP patch functions as a localized drug reservoir; when loaded with chemotherapeutics, it successfully suppressed the growth of peritoneal tumor colonies and was applied to regions at high risk for recurrence or metastasis after tumor resection. This multifunctional design underscores the IP patch’s potential as both a bioadhesive and therapeutic platform for managing complex intraperitoneal pathologies.

Bone repair in craniofacial defects is hindered by the oral cavity’s wet environment, which affects conventional hydrogel stability and performance due to frequent exposure to blood and saliva. Moshaverinia et al. [102] created a photoinduced in situ crosslinkable catechol-functionalized adhesive and osteoconductive hydrogel (AdhHG) with adjustable mechanical properties that can attach to bone surfaces even when saliva or other body fluids are present (Fig. 3b). The hydrogel exhibited good adherence to bone and soft tissue. Because of its good injectability and biocompatibility, the material was also used in tissue engineering as an MSCs carrier. In a therapeutic application for peri-implantitis, gingival mesenchymal stem cells were encapsulated within the hydrogel, resulting in effective regeneration of bone around damaged dental implants [102]. In another research, Zhang et al. [103] created an injectable, sticky hydrogel inspired by mussels that contains exosomes to promote endogenous cell recruitment and cartilage regeneration. A hydrogel exhibiting substantial adhesion to wet surfaces was synthesized through the formation of a crosslinked network comprising alginate-dopamine, chondroitin sulfate, and regenerated silk fibroin (AD/CS/RSF) (Fig. 3c). The hydrogel demonstrated significant bonding attachment to the wet surface, representing a promising minimally invasive strategy for treating cartilage abnormalities with arthroscopy help.

Another noteworthy marine-derived bioadhesive is the tunicate, an invertebrate organism characterized by its composition of highly crystalline cellulose nanofibers and protein components enriched with pyrogallol-containing amino acids, specifically 3,4,5-trihydroxyphenylalanine (TOPA). These TOPA residues endow the material with remarkable adhesive properties, enabling self-healing behavior in seawater. Analogous to catechol moieties, the gallol groups in TOPA are capable of forming covalent crosslinks through oxidative reactions or via coordination with metal ions [104,105].

## 4 Marine-derived lipids and pigments

Marine-derived lipids and pigments offer a wide range of biomedical applications. Fucoxanthin is a carotenoid pigment primarily found in brown algae [106,107]. Notably, fucoxanthin has demonstrated antioxidant, anti-inflammatory, and anticancer effects, making it a promising compound for therapeutic development [108]. In human keratinocytes, fucoxanthin reduces ROS levels, prevents DNA damage, boosts mitochondrial membrane potential, and inhibits apoptosis [109]. Yang et al. [110] demonstrate the potential of fucoxanthin in metabolic-associated fatty liver disease (MAFLD). It exerts its effects through the modulation of key molecular pathways involved in lipid metabolism, including the regulation of PPAR-α and SREBP-1c. Fucoxanthin activates AMPK, modulating the KEAP1/Nrf2/ARE pathway for antioxidative effects and stimulating the PGC1α/NRF1 axis to enhance mitochondrial biogenesis. These mechanisms help alleviate hepatic steatosis caused by metabolic disturbances, suggesting fucoxanthin’s potential as a therapeutic strategy for MAFLD [110]. Recent studies suggest that fucoxanthin may also contribute to metabolic health, specifically in the management of obesity and related conditions [111,112]. It exerts anti-obesity effects by promoting thermogenesis through mitochondrial uncoupling protein 1 (UCP1), converting white adipose tissue (WAT) into brown adipose tissue (BAT). It enhances lipid metabolism and mitigates myocardial damage, fibrosis, and hypertrophy. On the other hand, the consumption of fucoxanthin was able to lower blood glucose levels and plasma insulin.

Among the various lipids found in marine organisms, omega-3 fatty acids, particularly eicosapentaenoic acid (EPA) and docosahexaenoic acid (DHA), are well-known for their anti-inflammatory properties and health benefits, including cardiovascular and bone health [34,113]. Omega-3 fatty acids are commonly utilized as dietary supplements to reduce triglyceride levels and safeguard cardiovascular health. Ingestion of EPA and DHA significantly reduces the release of arachidonic acid from cell membrane phospholipids, inhibiting pro-inflammatory signaling pathways [114]. These omega-3 fatty acids decrease the production of pro-inflammatory mediators, including prostaglandins-2, leukotrienes, and hydroxyl eicosatetraenoic acid, by enzymes such as cyclooxygenase-2, lipoxygenase, and cytochrome P450, thus suppressing the inflammatory cascade. Additionally, EPA and DHA promote the production of resolvins, protectins, and maresins, which possess strong anti-inflammatory properties and aid in resolving inflammation. When incorporated into lipid nanocarriers, these PUFAs exhibit prolonged circulation time, reduced oxidative degradation, and improved drug delivery, enhancing efficacy and bioavailability while minimizing required doses compared to traditional fish oil supplements [115]. However, concerns regarding contaminants in marine animal co-products highlight the need for stringent quality control measures, such as hazard analysis and critical control point protocols, to ensure the safety and purity of lipid extracts [116,117]. With improvements in purification methods and sustainable sourcing, marine-derived lipids present a promising, bioactive option for lipid nanoparticles, thereby enhancing the efficiency of drug and gene delivery.

## 5 Current strategies for the fabrication of marine biomaterials

### 5.1 Hydrogels

Hydrogels, characterized by their three-dimensional crosslinked structures, serve as promising artificial ECM analogs in tissue engineering due to their high-water content, tunable physicochemical characteristics, compliant mechanical properties, capacity for in situ crosslinking, and efficient diffusion of bioactive substances [118,119]. Marine-derived hydrogels have the potential to be developed as an intelligent delivery system that may release large amounts of bioactive material at precise locations by reacting to internal body inputs (Table 2). Nonetheless, despite their beneficial features, conventional hydrogels often present drawbacks. Their lack of mechanical strength is a major disadvantage that limits their use in load-bearing tissues such as ligaments, tendons, cartilage, and bone. Pomeraniec et al. [120] prepared collagen fibers from coral that are embedded in alginate hydrogel to create a novel scaffold made of bio-composite materials that have been shown to exhibit improved mechanical qualities and promote MSCs proliferation. Alginate hydrogels exhibit moderate rates of breakdown, as mammalian cells lack the enzymes required to break down alginate. Furthermore, they have a limited capacity to adsorb proteins, which makes efficient cell attachment difficult. Therefore, the current work focuses on altering the alginate backbone chemically to improve biofunctionality and cellular interactions. Luo et al. [121] created a biocompatible, high-strength, and low-friction hydrogel by soaking PVA and chitosan in an aqueous solution of SA after three cycles of freezing and thawing. Its exceptional strength (highest compressive strength = 141 MPa) was due to crystalline domains, hydrogen bonds, and ionic interactions. Excellent cell compatibility was achieved through the use of biocompatible materials and green advancement. With all of these advantages, it was the perfect alternative to articular cartilage. Yu et al. manufactured an injectable, thermosensitive hydrogel utilizing hydroxypropyl chitin (HPCH), into which they added a combination of hyaluronic acid (HA) and salmon calcitonin (sCT) with a high association efficiency of 96.84 % [122]. Additionally, a similar technique was used to combine sCT with oxidized calcium alginate (OCA) to deliver bone-targeting nanoparticles. This osteoconductive hydrogel innovation offers an effective therapy for bone-related disease [123].

**Fig. 4:**
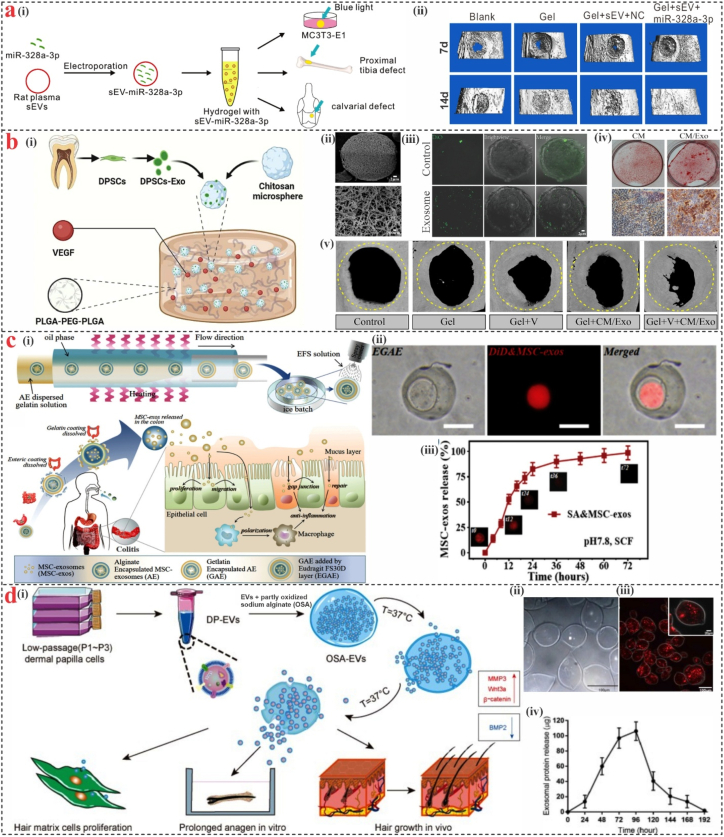
**Marine-derived hydrogels.** (**a)** (i) Schematic representation of EVs encapsulated into methacrylated glycol chitosan hydrogels for bone defect treatment, (ii) 3D Micro-CT images of the rats proximal tibial defect areas after they received hydrogels containing EVs [124]. (**b)** (i) Injectable microsphere-based hydrogel schematic representation for sequential release of DPSCs-Exo and VEGF; for enhanced bone regeneration, (ii) SEM image of chitosan microsphere, (iii) DiO-labeled DPSCs-Exo on distribution on microsphere, (iv) ARS staining after 21 days, (v) Micro-CT of calvarial defects at 8 weeks, yellow dashed line indicating defect area [127]. (**c)** (i) Schematic of gelatin microcapsules with alginate-encapsulated MSC-Exo, (ii) Optical and fluorescent images of DiD-labeled MSC-Exo at pH 7.8, 37 °C, (iii) Release profile and fluorescence images at different time points [128]. (**d)** (i) Diagram of OSA-EV nanospheres and their function, the extended release of DP-EVs from OSA-EV microgels led to in vivo hair growth, prolonged anagen in vitro, and cell proliferation, (ii) bright field (iii) and confocal images of OSA-EV microgels with DIL-labeled DP-EVs, (iv) in vitro release of vesicular proteins [129]. (For interpretation of the references to color in this figure legend, the reader is referred to the Web version of this article.)

Currently, marine-derived hydrogel systems are a promising option for the delivery of EVs. According to a study by Xia et al., a methacrylated glycol chitosan hydrogel injected into sEVs with miR-328a-3p may effectively improve bone healing by targeting osteoprogenitors (Fig. 4a) [124]. In contrast to traditional surgical implantation, hydrogel microspheres have recently gained popularity due to their more controlled size, less invasive injection method, enhanced specific surface area and porosity, ease of surface modification, and efficient loading of seed cells or bioactive substances/drugs [125]. Hydrogel microspheres are a superior option for healing bone abnormalities because they can be made to fit a variety of irregular wounds [126]. Han et al. [127] created an injectable thermosensitive hydrogel that contained chitosan microspheres loaded with dental pulp stem cell-derived exosomes (DPSCs-Exo) and vascular endothelial growth factor (VEGF) (Fig. 4 b). DPSCs-Exo were released gradually to facilitate long-lasting bone repair, and quickly released VEGF, encouraging the development of new blood vessels. The creation of new bone tissue inside the defects was aided by the VEGF and DPSCs-Exo controlled release.

Gan et al. [128] initially incorporated MSC-EVs in SA hydrogel microspheres to preserve their activity for oral medication delivery in inflammatory bowel disease (Fig. 4c). To protect the MSC-EVs microspheres from the acidic environment of the stomach, the gelatin shell was added to the MSC-EVs loaded SA core microspheres. The results showed that the oral MSC-EVs encapsulated microspheres may have potential clinical value for various diseases. Additionally, marine-derived hydrogel microspheres have been applied to hair regeneration. For local injection, Chen et al. [129] covered human dermal papilla cell-derived EVs in a partly oxidized sodium alginate oxalate (OSA) hydrogel (Fig. 4d). Results showed that OSA-EVs significantly increased the growth of hair stromal cells, extended the primordial phase of cultured human hairs, and sped up the regeneration of dorsal hairs in mice following hair removal. The increase of signaling molecules that promote hair development, like Wnt3a and β-catenin, and the downregulation of the inhibitory molecule BMP2 may be the cause of these effects.

### 5.2 3D bioprinting

**Fig. 5:**
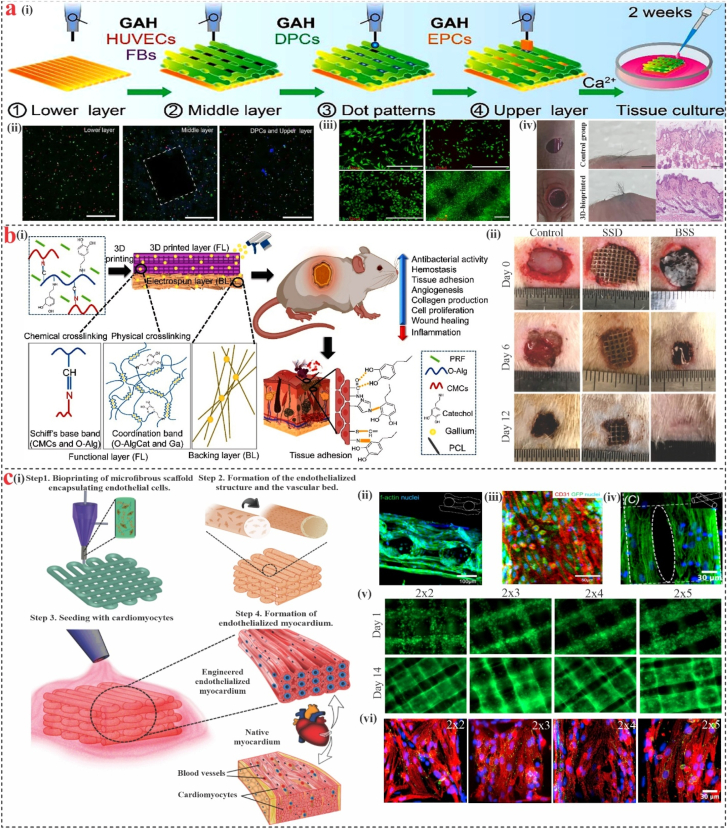
**Marine-derived 3D bioprinting.(a)** (i) Schematic overview of the 3D bioprinting process, (ii) cell distribution in the scaffold, (iii) The viability of FB, EPC, and HUVECs in 2D cultures and 3D-bioprinted scaffolds was assessed by live/dead staining, (iv) 3D‐bioprinted multilayer scaffolds for HFs reconstruction in vivo. Reproduced with permission [134]. **(b)** (i) An illustration of the multifunctional bilayer skin replacement (BSS) internal structure and process, (ii) in vivo evaluation of wound healing efficacy in infected burn models, comparing BSS, commercial, and untreated control [135]. **(c)** (i) Diagrammatic representation of the 3D bioprinting method used to create endothelialized myocardium, (ii) confocal microscopy image of a tri-layered scaffold, indicating HUVECs-induced endothelial layer development, (iii) GFP-HUVECs in a single fiber for CD31, GFP, and nuclei, (iv) F-actin staining reveals the arrangement of cardiomyocytes on scaffold surfaces, notably at the intersections of neighboring microfibers, (v) Spatial distribution and spreading patterns of GFP-HUVECs within microfibrous scaffolds with varying aspect ratios, (vi) Immunofluorescence analysis of connexin-43 (Cx-43, green) and sarcomeric α-actinin (red), showing variations in alignment in cardiomyocytes implanted on scaffolds with various geometric shapes [136]. (For interpretation of the references to color in this figure legend, the reader is referred to the Web version of this article.)

In the recent decade, 3D printing has been widely used to investigate and produce medical products such as prostheses, biodegradable metal implants, bone/cartilage substitutes, and even living tissues and organs [130,131]. 3D bioprinting with marine-derived biopolymers offers additional benefits over synthetic materials, including self-assembling ability and similarity to the composition or structure of the ECM (Table 2). Alginate from brown seaweed is extensively applied in 3D printing, and many ways are adopted to overcome its limitations, such as mixing it with nanoclay or nanocellulose to improve printability and mechanical qualities [132]. Kosik et al. [133] designed a composite bioink for 3D-printed hydrogels by incorporating short submicron polylactide (PLA) fibers into an alginate matrix. The addition of PLA fibers significantly enhanced the mechanical characteristics of the constructs, increasing the Young’s modulus from 6.9 kPa to 25.1 kPa. In vitro studies confirmed that human chondrocytes maintained their viability (∼80 %) and structural integrity within the 3D-printed hydrogel filaments over a two-week culture period. Furthermore, it has been demonstrated that hydrogel dressings made via 3D printing, which combine biomaterials, bioactive substances, and living cells, have been shown to support the regeneration of different appendages. Kang et al. [134] created a multilayer composite scaffold by embedding fibroblasts, HUVECs, dermal papilla cells (DPCs), and epidermal cells (EPCs) in 3D-printed Gel/Alg gel (Fig. 5a). The bioprinted scaffold was then placed into full-thickness wounds in naked mice, exhibiting a structure similar to that of the epidermis and dermis. ALP, β-catenin, and α-SMA, DPC genes linked to hair induction, were restored, and self-aggregating DPC spheroids were made easier to form.

Due to limitations resulting from structural uncertainty and the complexity of physiological properties, classical tissue engineering substitutes cannot fully replicate the organism’s natural skin. Utilizing 3D extrusion printing, Khalaji et al. [135] demonstrated a novel 3D scaffold. They created a BSS by customizing carboxymethyl chitosan (CMCs) and oxidized alginate grafted catechol (O-AlgCat) on a hydrophobic electrospun layer (Fig. 5b). In a model of an infected rat burn, the dressing demonstrated good anti-inflammatory effects, lowering the expression of proinflammatory factors and accelerating the healing process. Another study used extrusion technology to print SA, EVs, and hyaluronic acid bio-ink layer by layer. The bio-ink was then cross-linked using CaCl_2_ to enhance the interfacial adhesion between the layers and sustained release of EVs. Through the regulation of the inflammatory response, the system may improve tissue repair [137].

The layered arrangement enhanced the mechanical performance of marine 3D-printed materials. Ji et al. [138] created a multilayered scaffold for bone regeneration that was printed in three dimensions, taking inspiration from the adhesive mechanisms of mussels. The scaffold exhibited favorable mechanical properties and possesses potential osteogenic and antibacterial capabilities. Zhang et al. [136] demonstrated a new hybrid approach that relies on 3D bioprinting (Fig. 5c). In order to create a layer of confluent endothelium, endothelial cells were first directly bioprinted inside microfibrous alginate and GelMA hydrogel scaffolds. After that, human cardiomyocytes with the capacity to contract were created by seeding them on 3D endothelial scaffolds. Additionally, an endothelialized myocardium-on-a-chip system for assessing cardiovascular toxicity was established by integrating the organoids into a specially created microfluidic perfusion bioreactor. The results showed that this method could be applied to human cardiomyocytes produced from induced pluripotent stem cells to create endothelialized human myocardium.

One of the exciting innovations in the field is coaxial bioprinting, which enables the simultaneous extrusion of multiple biomaterials to form core-shell structures. This technique can improve the functional and mechanical properties of the resulting constructs. Liu et al. [139] fabricated polydopamine (PDA) coated Alg-Gel/PCL core–shell scaffolds to enhance their photothermal reaction. Upon exposure to NIR light, the PDA layer generated localized heat, inducing a sol-gel transition in the core hydrogel and causing the drug (adriamycin) to be released on demand. This technique has a lot of potential for use in encouraging tissue regeneration and fixing tissue defects.

### 5.3 Artificial intelligence and machine learning-assisted 3D printing

The integration of Artificial Intelligence (AI) and Machine Learning (ML) with marine-derived biomaterials has significantly transformed the biomedical field, enabling faster, more efficient, and more precise innovations in areas like wound healing, tissue regeneration, and drug delivery systems. AI plays a pivotal role in advancing biomaterial development for 3D bioprinting by facilitating three core functions: forecasting the mechanical and physicochemical characteristics of materials, enabling the intelligent selection of appropriate biomaterials, and optimizing printing parameters to improve fabrication efficiency and accuracy [140,141]. The creation of intelligent dressings with real-time wound monitoring ability allows for feedback-based treatment strategy adjustments, providing targeted treatments and fostering the best possible tissue recovery. AI must be integrated into the manufacturing system to detect, adjust, and anticipate the dynamic conditions of the printing environment, such as morphologically changing objects.

**Fig. 6:**
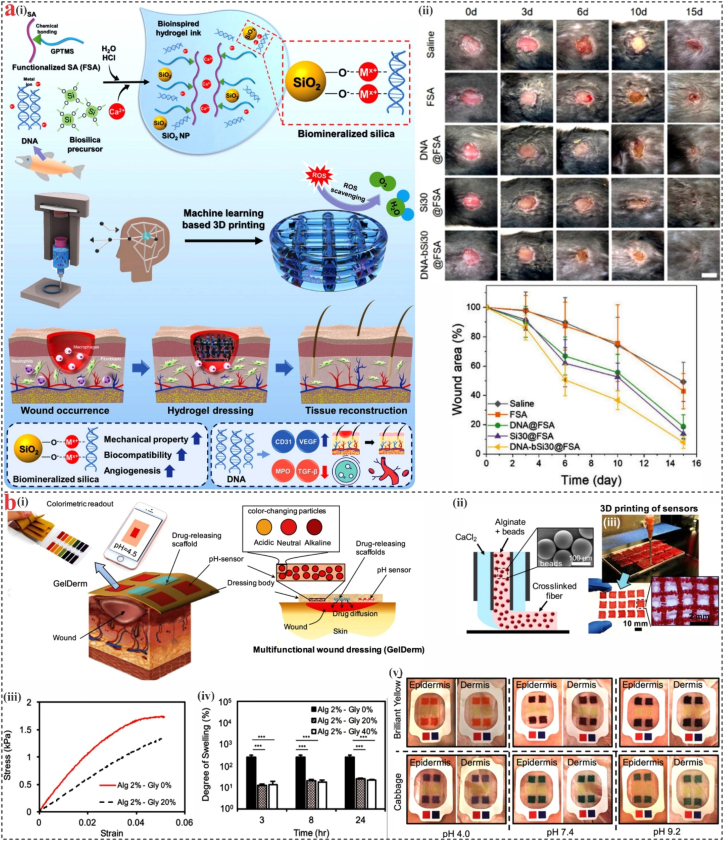
**ML and AI monitoring-3D bioprinting. (a)** (i) Schematic illustration of the construction process of ML-guided 3D-printed hydrogel structures, (ii) Images of wound areas after specified healing duration. Reproduced with permission [146]. **(b)** (i) GelDerm application with pH-sensitive and drug-releasing components, (ii) Schematic of the co-axial fiber deposition system, (iii) Stress-strain graph from pure alginate and alginate–glycerol blend dressing, (iv) Glycerol incorporation notably decreased the swelling behavior of the hydrogel, (v) Photographs of GelDerm/Mepitel dressings applied to pig dermis and epidermis treated with solutions of varying pH. Reproduced with permission [147].

ML analyzes and precisely forecasts experimental results by applying model parameters that have been learned from training datasets. ML offers a dependable method for gene sequencing, protein structure prediction, and disease diagnosis in biomedicine [[142], [143], [144], [145]]. In a study by Kim et al. [146], ML-assisted optimization of 3D printing parameters enabled the construction of hydrogel dressings with high accuracy, structural fidelity, and tunable mechanical properties, allowing for customized treatment of wounds with varying shapes and depths (Fig. 6a). Building on this, functional hydrogel inks incorporating salmon sperm-derived DNA and DNA-induced biosilica, bioinspired by marine sponges, have been developed for ML-guided 3D printing. These bioinks not only exhibit excellent printability and porosity but also enhance biological functions such as reactive oxygen species scavenging, angiogenesis, and anti-inflammatory activity. The resulting dressings significantly improve healing in both acute and diabetic wounds, offering a promising marine-derived platform for precision wound care. According to studies, the pH value of the wound microenvironment fluctuates dramatically during wound healing; hence, pH monitoring is a reliable and helpful tool. Mirani et al. [147] offered a sophisticated multi-functional alginate dressing including antibiotics (gentamicin sulfate) to boost the intelligent effect of dressings. The dressing can analyze pH levels colorimetrically, detect bacterial infections, and release medications on the wound (Fig. 6b). Additionally, the dressing can be integrated onto a commercial patch and wirelessly connected to a smartphone to enable the sharing of digital image data about the wound between patients and healthcare professionals. It offers a wide range of potential applications as an intelligent dressing for both acute and chronic wounds. In a study by Chen et al. [148], an AI-assisted high-throughput system was developed that integrates a programmable pneumatic extruder and image-based analysis to optimize bioprinting parameters. By varying printing pressure, speed, and nozzle distance using an inclined surface, the system efficiently mapped printable conditions for alginate/gelatin-based bioinks. This approach enabled the identification of printing settings that enhanced both mechanical integrity and biological performance. The methodology shows promise for broader use in optimizing extrusion printing of temperature-sensitive marine-derived biomaterials.

Intelligent 3D-printed hydrogel dressings, equipped with integrated biochemical sensors and data transmission units, enable real-time monitoring of wound conditions and visual output across media platforms, offering significant advantages for remote healthcare and personalized wound management. AI-assisted image analysis is proving essential in optimizing 3D printing parameters [149]. The creation of electronic skin (e-skin) that replicates the complex functions of human epidermis and dermis is in high demand for applications in human–machine interaction [150]. As the demand for precise, structurally complex, and rapidly fabricated biomaterials increases, AI’s capacity to process and analyze large datasets becomes critical for adapting natural marine-derived materials to advanced biomedical applications.

### 5.4 Electrospinning

The electrospinning method, recognized for its facile fabrication of nano-to microscale 3D-porous structures, has garnered considerable interest for the development of drug-loaded fibers. This technique offers notable advantages with marine-derived materials, including (a) the ability to regulate the initial burst release of therapeutic agents and (b) the possibility of encapsulating a wide range of biomolecules (Table 2) [151,152]. Chen et al. [153] created core-shell electrospun fibers made of gelatin and chitosan, and they used a wet chemical process to deposit hydroxyapatite on their surfaces. The fibers were fabricated using coaxial electrospinning, designed to replicate the structure of bone ECM. In vitro evaluations demonstrated improved osteoblast proliferation, indicating their potential for bone tissue engineering. Additionally, kappa-carrageenan is recognized as a promising biopolymer for producing degradable nanofibers applicable in tissue engineering [154]. Alizadeh et al. [155] fabricated a bilayer wound dressing consisting of a gelatin sponge cross-linked with sodium tripolyphosphate (Gel-STPP) and a carrageenan nanofiber layer embedded with platelet-rich fibrin (Carr-PRF). The incorporation of the Carr-PRF layer enhanced the mechanical properties, increasing the tensile strength by 12.96 % (from 0.216 to 0.268 MPa) and the elastic modulus by 56.70 % (from 0.388 to 0.608 MPa) relative to the Gel-STPP alone. After 14 days, Gel-STPP/Carr-PRF dressing accelerated wound healing and the formation of the keratin layer and skin appendages. Another current study used κ-carrageenan to create a biocompatible scaffold for bone tissue regeneration by blending it with biodegradable polyesters. In contrast to the micron-sized apatite crystals that formed on the surface of the pure polyesters, the existence of κ-carrageenan at the blend fiber surface produced nano-sized ones. The inclusion of κ-carrageenan into polyhydroxybutyrate and polyhydroxybutyrate valerate fibers led to better mineralization and differentiation of SaOS-2 in vitro [156]. The nanofiber scaffold made by combining fish gelatin with additional chemical ingredients was also shown to be a viable model for 3D cell culture. It not only supports cells but also promotes protein and gene expression that are essential for cell adhesion and development [157]. Chandika et al. [158] prepared a bilayer nanofibrous scaffold consisting of fish collagen and PCL. Cytocompatibility analysis demonstrated that scaffolds with a high fish collagen content exhibited functional activity in promoting normal responses from HaCaT keratinocyte cells and human dermal fibroblast neonatal (NHDFneo), suggesting that they could be useful tissue-engineered implants for full-thickness wound healing applications [158]. One of the most frequent injuries to the skin is a burn, which may be associated with significant morbidity and mortality depending on the extent of the damage. Terezaki et al. [159] created electrospun matrices based on ulvan and gelatin, which they used as wound dressings for the swollen, burnt skin of female SKH-1 hairless mice. The outcome makes it clear that ulvan/gelatin-nanofibrous patches are a potential treatment for burn wounds. It was demonstrated that the manufactured ULV/PEO/GEL (2:1:1) and ULV/PEO/GEL (1:1:2) patches were effective treatments for burn wounds because they accelerated wound contraction in the early phases of the healing process, reduced inflammation, and produced uniform wound closure [159].

**Table 2:** Several examples of marine-inspired materials led over the last ten years using different fabrication methods.

Marine material	Fabrication technique	Cell-Line	Characterization methods	Results	Ref.
Alginate	Hydrogel	L929	TEM, photothermal behaviors, cytotoxicity assessment, antibacterial activity	Inhibits the bacteria colonization, promoting angiogenesis and collagen deposition, and accelerating wound closure.	[160]
Chitosan	Hydrogel	L929	^1^H NMR, FTIR, SEM, rheological and mechanical test, hemolytic and cytocompatibility test	Antibacterial and antioxidant functions promoted wound healing.	[161]
Chitosan	Hydrogel	HUVEC	XRD, TEM, hemolysis, cytocompatibility, and antibacterial test	Hydrogels promoted skin regeneration by upregulating collagen, STAT3, and VEGF expression, reducing TNF-α and IL-6 expression, and stimulating epithelial and dermal regeneration.	[162]
Alginate	Bioprinting	NIH 3T3	Mechanical and rheological measurements, and Cell proliferation assay	Excellent growth and viability of the printed cells up to 7 days of culture.	[163]
Shark skin collagen	Bioprinting	L929	FT-IR, XRD, TEM, TGA, rheological properties, DNA quantification, and metabolic activity	Induce a favorable cellular response to direct cellular activity.	[164]
κ-Carrageenan	Bioprinting	L929	Mechanical compression, swelling degree, contact angle, rheological characterization, and biocompatibility assays	Structures maintained high cell survival for up to 11 days in cell culture environments without the need for sacrificial supporting materials.	[165]
Chitosan	Bioprinting	MSCs	SEM, TEM, FTIR, adsorption efficiency, and cytocompatibility	Stimulates cartilage regeneration and offers a favorable milieu for chondrogenic differentiation and proliferation.	[166]
Fish collagen	Electrospinning	HdFa, NIH-3 T3	SEM, FTIR, TGA, water contact angle, and MTT assay,	Fish collagen supported the growth and proliferation of the fibroblast cells.	[167]
Chitosan	Electrospinning	hBMSC	SEM, TEM, dynamic light scattering assay, bioactivity assay, RT-PCR, histological, and immunohistochemical staining	Preloading Nel-like molecule-1 growth factors into chitosan nanoparticles significantly delayed the release time and improved the expression of chondrogenic-related genes and proteins.	[168]
Alginate	Electrospinning	HEK-293T	SEM, FTIR, water contact angle, cytotoxicity assay, and in situ transfection experiments	Composite nanofibers enhance cell survival and deliver target genes to adherent cells, and can be used for regenerative gene therapy.	[169]

## 6 Marine biomaterials in tissue engineering and regenerative medicine

### 6.1 Bone tissue engineering

**Fig. 7:**
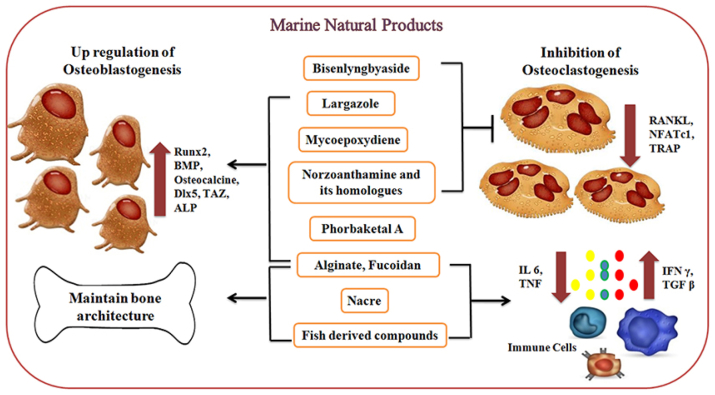
Marine-derived materials impact on bone metabolism through osteoblastogenesis upregulation and osteoclastogenesis downregulation, reproduced from Ref. [171].

**Fig. 8:**
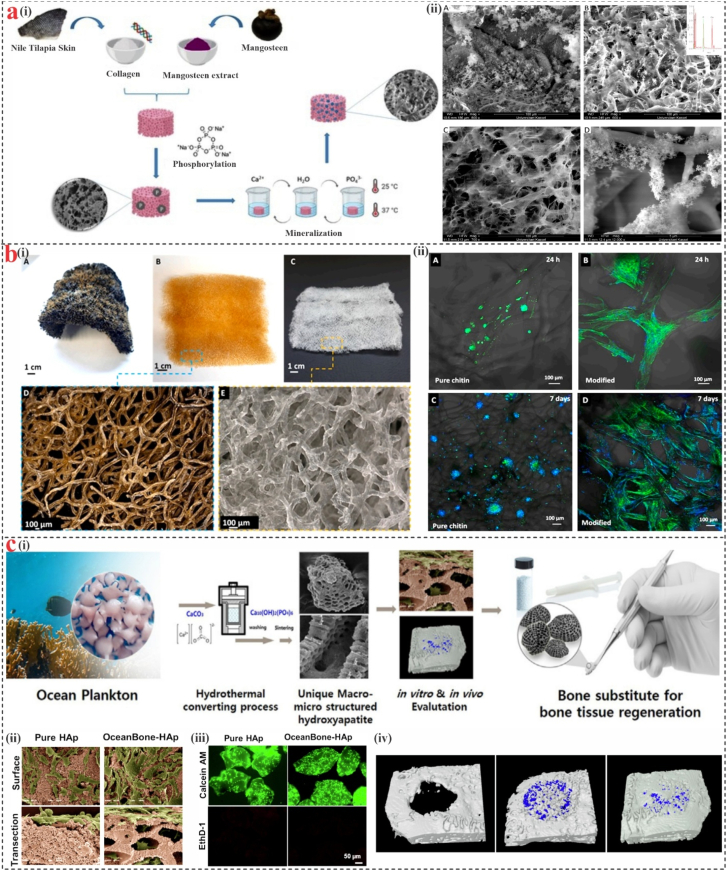
**Examples of marine-derived compounds for bone repair and regeneration.** (a) (i) Preparation of phosphorylated fish skin collagen/mangosteen scaffolds, (ii) SEM and EDX analysis of collagen scaffold and calcium phosphate mineralization [172]. **(b)** (i) Preparation of decellularized and demineralized flexible skeleton containing bromide-rich pigment in microtubular fibers, (ii) F-actin filament morphology and organization of hFOB 1.19 cells cultured on pristine and biomineralized chitinous scaffolds after 24 h and 7 days showed aggregate formation and extensive cell spreading [173]. **(c)** (i) Schematic illustration of honeycombed HAp bone granules derived from marine plankton exoskeletons, (ii) SEM image of hMSCs cultured on pure HAp and OceanBone-HAp, (iii) Cytotoxicity evaluation of OceanBone-HAp extract, (iv) Micro-CT images following 8-week implantation [175].

The treatment of bone-related defects and diseases is a serious concern in modern clinical practice. The need for bone biomaterials has grown significantly in recent years. The structural and functional properties of materials must be tailored to the location of the defect and the patient’s physiological state. The design of materials applied for bone tissue engineering (BTE) mostly presents a combination of porous structures that support vascular and cellular migration with the multiscale structure and order of the bone matrix [170]. Marine-derived compounds, such as alginate, fucoidan, phorbaketal A, largazole, and norzoanthamine, play a direct role in modulating the levels of osteoblastic markers Runx2, BMPs, osteocalcin, and other transcription factors. The production of cytokines IL6 and TNF is controlled by nacre proteins from mollusks and compounds from fish, which have been demonstrated to enhance biomineralization and inhibit bone resorption activity. In contrast, marine-derived substances, including mycoepoxydiene, norzoanthamine, and its homologs, biselyngbyaside and largazole decreased RANKL-induced osteoclastogenesis through regulation of the RANK/RANKL/osteoprotegerin pathway (Fig. 7) [171]. Numerous marine biomaterials for bone regeneration have been investigated recently in this context. Eduardo et al. [172] extracted collagen and mangosteen from Tilapia fish skin and waste peel of the respective fruit (Fig. 8a). The scaffolds were phosphorylated with sodium trimetaphosphate to enhance nucleation sites for mineralization. Mineralization was successful, and SEM revealed the presence of calcium phosphate. Investigations on phosphorylated fish collagen using extract from mangosteen peel showed that the generated mineralized scaffolds were promising materials for BTE and a novel technique. Further research is needed to assess the material interaction with cellular adhesion and tissue regeneration, particularly in vivo. Biofunctionalization is a powerful strategy to improve scaffold surface characteristics by altering particular attributes such as topography, mechanical behavior, wettability, surface roughness, and functional group charge. This modification plays a critical role in regulating cellular adhesion, growth, and differentiation. Machałowski et al. [173] worked on biofunctionalized chitin scaffolds for BTE (Fig. 8b). An ex vivo biomineralization approach was used to functionalize 3D chitinous scaffolds obtained from the marine verongiid demosponge, Aplysina fistularis. The human fetal osteoblast cell line hFOB 1.19 was examined for the first time to assess the biological potential of the newly produced. Mechanical and atomic force microscopy (AFM) investigations have demonstrated that biofunctionalized scaffolds exhibit a mechanical resistance approximately four times greater, accompanied by topographical alterations, including an increase in surface roughness (Rq) from 31.75 nm to 120.7 nm.

As a next-generation implant material, biogenic hydroxyapatite synthesis from marine sources was recently introduced. Its significant porosity design and mineral ions facilitated biocompatibility and bone bonding capabilities [174]. Baek et al. [175] worked on a bone transplant substitute made from ocean planktonic exoskeletons (OceanBone-HAp) (Fig. 8c). In vitro analysis revealed that OceanBoneHAp, in contrast to stoichiometric synthetic HAp granules, enhanced cellular attachment, differentiation, infiltration, and growth. Furthermore, qPCR proved that OceanBone-HAp enhanced the expression of osteogenic markers, such as ALP, Col1a1, OCN, and BSP. The material’s efficacy in promoting bone regeneration was further validated using a rabbit calvarial defect model. The findings demonstrated that OceanBone-HAp significantly increased the area of mature bone formation compared to pure HAp. Nevertheless, the need for porosity in nano-hydroxyapatite to improve cell attachment with these scaffolds has led to further processing using high-energy machinery or synthetic chemicals, which could increase the cost of manufacture and cytotoxicity. The capacity of biomolecular extracts from marine sponges to mimic biological processes has recently garnered significant attention for the creation of silica-calcium composites. Furthermore, it was suggested that the distinct structure of marine sponges could be a superior substitute for traditional scaffolds [176].

### 6.2 Skin tissue engineering and wound healing

**Fig. 9:**
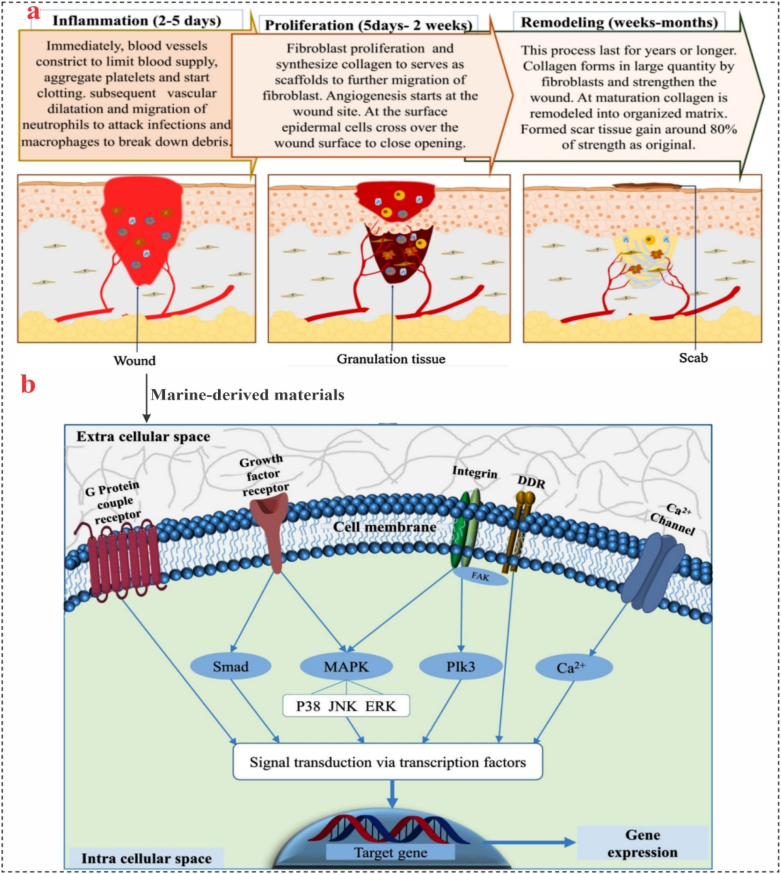
**Marine-derived biomaterials promise in wound healing and skin regeneration. (a)** Illustration of wound healing process, **(b)** An overview of chemical and mechanical signal transduction pathways. The developing trend in marine materials facilitates faster wound healing and the construction of native skin tissue at different stages via cellular-level activation. Reproduced from Ref. [177].

The need for skin replacements to repair skin abnormalities caused by burns, trauma, infection, graft rejection, scarring, hereditary flaws, and other disorders has grown over the centuries, posing a significant healthcare challenge. Typically, the healing of a wound injury in humans can be divided into three phases, as follows: inflammation (acute and chronic), proliferation (neo-tissue formation), and tissue remodeling (Fig. 9a) [177]. Marine biomaterials are essential for skin tissue regeneration and wound healing (Fig. 9b).

**Fig. 10:**
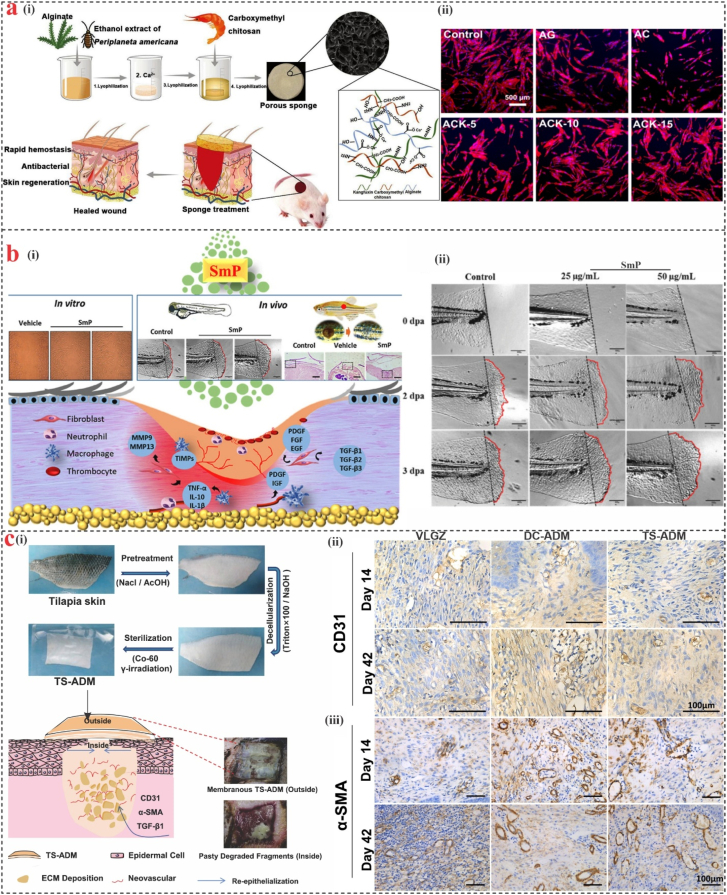
**Examples of marine-derived biomaterials for wound healing and skin repair. (a)** (i) Schematic illustration of the ACK composite sponges for full-thickness skin repair, (ii) Fluorescence imaging of hGF response after different sponge formulations [180]. **(b)** (i) Schematic illustration of marine algae SmP potential in regenerative and wound-healing agent, (ii) Influence of SmP treatment on caudal fin regeneration in larval zebrafish in the control group and different treatment groups (25 and 50 μg/mL) [182]. **(c)** (i) Schematic representation of TS-ADM fabrication and its partial degradation, which facilitates ECM deposition, angiogenesis, and re-epithelialization by supporting the expression of TGF-β1, α-SMA, and CD31, (ii) Immunohistochemical images of CD31, and (iii) α-SMA expression [184].

Several studies have proved that marine-derived collagen can significantly enhance wound healing, a potential dermal substitute for the treatment of severe wounds. Zhang et al. [178] found that collagen peptides extracted from Nile tilapia (*O. niloticus*) skin exhibited effective therapeutic potential in burn wound repair. Ultimately, a variety of marine hydrogels have been developed to accelerate wound healing, including marine collagen derived from *Oncorhynchus keta, Lates calcarifer, Stichopus japonicas, Salmo salar,* and *Stomolophus nomurai meleagris;* alginate derived from *Macrocystis pyrifera*; and chitosan derived from crabs and shrimps [179]. Yun et al. [180] created a biodegradable antibacterial sponge by combining a traditional Chinese medication called kangfuxin (KFX) with a composite of alginate (AG) and carboxymethyl chitosan (CMC) through green crosslinking, electrostatic interaction, and freeze-drying methods (Fig. 10a). According to these findings, the AG/CMC/KFX (ACK) sponges have a highly porous and interconnected structure, good elastic qualities, appropriate water vapor transmittance, antibacterial activity, cytocompatibility, and quick hemostasis. In addition, the ACK sponge with 10 % KFX (ACK-10) considerably facilitated wound closure in a rat wound model.

Chronic hyperglycemia, tissue hypoxia, inflammatory responses, and severe vascular lesions are among the many variables that make it difficult for people with diabetes to treat their wounds, making them difficult to heal normally. Carrageenan oligosaccharides have been shown to stimulate the secretion of anti-inflammatory mediators in murine blood. Song et al. [181] synthesized a low-sulfation κ-carrageenan oligosaccharide (L-DS-KOS) by a desulfation process that involved DMSO-methanol and developed a sponge dressing for diabetic rat wound healing. The results showed that L-DS-KOS could efficiently stimulate the release of anti-inflammatory substances and accelerate the polarization of macrophages from M1 to M2. Edirisinghe et al. [182] used a zebrafish model for an in vivo study and human dermal fibroblasts (HDFs) for in vitro experiments to examine the regenerative and wound-healing properties of Spirulina (Arthrospira) maxima-derived pectin (SmP) (Fig. 10b). When compared to untreated HDFs, SmP-treated (12.5–50 g/mL) HDFs boosted cell growth by 20–40 %. Therefore, SmP may be used as a possible agent for wound healing and regeneration.

In order to create particular scaffolds with good swelling characteristics as well as hemostatic and antibacterial capabilities, chitosan can be added directly to wound healing materials or combined with other composite materials. One of the primary reasons for chitosan’s popularity in wound healing is its potent antibacterial action against *Gram-positive* and *Gram-negative* bacteria and fungi [183]. In another study, Li et al. [184] created a novel acellular dermal matrix (TS-ADM) from tilapia skin using alkaline decellularization and γ-irradiation methods (Fig. 10c). TS-ADM differs from commercial pig acellular dermal matrix (DC-ADM) in terms of water vapor transmission, biodegradability, thermal stability, and shape. In this case, innovative TS-ADM could create a microenvironment that is favorable for wound healing when utilized as an inexpensive bioactive dressing. Research interest in novel wound dressings made from marine biomaterials are gaining popularity, especially in light of the acidic pH, high glucose, and high reactive oxygen species milieu of diabetic wounds.

### 6.3 Marine biomaterials application in other diseases

Marine biomaterials have garnered attention in liver development, anticancer therapies, connective tissue repair, and central nervous system repair mechanisms. It is anticipated that SA will be utilized as a pH-responsive hydrogel for logical drug release in certain specific contexts due to its high pH sensitivity and ability to quickly form gels under highly mild conditions through ion exchange reactions with cations [185]. Additionally, alginate can be employed as a cell-loading carrier [186,187]. Liao et al. [188] designed microspheres by embedding adipose-derived stem cells (ADSCs) in alginate-gelatin (Alg-Gel). ADSCs embedded in Alg-gel microspheres exhibit exceptional healing capability and have potential for use in the restoration of cartilage tissue. Muthulakshmi et al. [189] design carrageenan as a reduction and capping agent for silver nanoparticles (AgNPs), and combined with gelatin hydrogel. Analysis was done on its antibacterial activity against several human pathogens, including *S. agalactiae 1661*, *S. pyogenes 1210*, and *E. coli*. This study demonstrated the prospect of using Ag/carrageenan-gelatin hybrid hydrogels for drug delivery, antibacterial, and anticancer applications [189].

The unique sulfation patterns within CS and DS polysaccharide chains have a significant impact on their interactions with bioactive proteins involved in the proliferation of hepatocytes and neural stem cells [190]. Oral medication delivery methods that target CD44-overexpressed colonic macrophages through receptor-mediated endocytosis could use CS. A multifunctional drug delivery system was achieved by surface coating a mesoporous MnO_x_ loaded with indocyanine green derivatives with CS and then embedding it in a chitosan/alginate hydrogel containing silk fibroin. This method was preferentially internalized by colon carcinoma cells by CS-mediated CD44 targeting after oral treatment. The payloads were then released through the response breakdown of silk fibroin. Using a combination of immune checkpoint inhibitors and direct synergistic tumor suppression, the released Mn^2+^ and the preferentially gathered Mn^2+^ around mitochondria catalyzed endogenous H_2_O_2_ into toxic OH through Fenton-like reactions, providing dual-targeted treatment of colon tumors [191]. HA interacts with specific cell surface receptors, triggering intracellular signaling cascades that regulate diverse cellular activities, including survival, migration, differentiation, and proliferation [192]. Notably, HA and the CD44 receptor can interact to activate key signaling pathways, including mitogen-activated protein kinases (MAPKs) and phosphoinositide 3-kinase (PI3K) [193,194]. The potential of fish gelatin to release active components into the oral cavity and administer medications orally is also being studied [195].

Ulvan improves the diversity of important key microbial species within the gut microbiota and activates their metabolic process, including glycolysis, the pentose phosphate pathway, and histidine/lysine biosynthesis. These processes support better gut health and metabolic balance by increasing the synthesis of short-chain fatty acids and activating AMPK. Through the MTF1/PPARα pathway, ulvan oligosaccharide also significantly lowers body and adipose tissue weights by reversing fatty acid β-oxidation and cholesterol transport [196]. Berri et al. [197] employed Ulva armoricana to boost the gut’s immune response using an in vitro model of porcine intestinal epithelial (IPEC-1) cells. U. armoricana ulvan stimulates the immune system by interacting with TLR4, activating mRNA expression of IL-8, TNF-α, and CCL20, and activating the NF-κB and PI3K/Akt signalling pathways [197].

Numerous investigations have shown that fucoidan protects the function of liver and kidneys in both human and diabetic animal models [198,199]. Tsai et al. were the first to disclose that a multifunctional nanoplatform could be developed by combining trimethyl chitosan (TMC) with fucoidan. At a dosage of 2 mg/mL, this system achieved a 33.2 % inhibition rate of α-glucosidase activity while simultaneously improving the transepithelial transport of insulin over the intestinal epithelial barrier [200]. Furthermore, it has been demonstrated that fucoidan either supports the innate immune response or is immunologically inert [201,202]. Yao et al. [203] demonstrated that adding fucoidan to PVA and utilizing sodium trimetaphosphate (STMP) to co-crosslink the composite improved endothelial cell adhesion and maintained monolayer functioning. As demonstrated by both in vitro and ex vivo investigations, the fucoidan-modified PVA showed inhibition of thrombin production and decreased platelet adhesion and activation. Additionally, in vivo evaluations utilizing a rabbit vascular graft model demonstrated a notable decrease in the development of intimal hyperplasia, increased endothelialization, and better graft stability [203]. Shanmugapriya [204] created a composite hydrogel based on fucoidan that contains collagen and nanohydroxyapatite (nHA) for targeted drug delivery to cancer cells in the GI. According to the study, the hydrogel had smaller pores, which improved biocompatibility, boosted cellular motility and proliferation, and decreased intracellular reactive oxygen species and singlet oxygen production [204]. Additionally, fucoidans are also recognized for their anticancer potential, primarily through the modulation of apoptosis in cancer cells [205]. Their antitumor activity is intimately linked to the degree of sulfation, the specific connections between sugar residues, and the makeup of monosaccharides. Several marine-derived polysaccharides are well-regarded for their bioadhesive properties, which enhance their ability to adhere to mucosal surfaces and epithelial tissues. This characteristic significantly improves transmucosal transport and facilitates more efficient absorption of therapeutic agents [[206], [207], [208]]. In summary, the exceptional properties of marine materials should be investigated and employed in a diverse array of biomedical disciplines (Table 3).

**Table 3:** Quantitative evidence supporting the advantages of marine-derived biomaterials (2019–2025).

Material	Source	Mechanical strength	Degradation	Biocompatibility	Outcome	Ref.
**Collagen**	Fish skin, scales, jellyfish, blue shark skin, and teeth	Young’s modulus of fish skin ∼5.76 MPa, and fish scale ∼2.43 MPa	Good degradation ability in approximately 20 days	Excellent biocompatibility, antioxidant activity, and antibacterial activity with no hemolysis	Suitable for wound healing application. Higher tendency for new bone tissue formation, 17.9 % compared to bovine, 12.9 %.	[[209], [210], [211], [212], [213]]
**Collagen Type I**	Tilapia fish skin	Tensile stress of 4.12 MPa	Slower degradation at 8 weeks after post-implantation	Lower immunogenicity with better compatibility than bovine COL-I	An appropriate implantable property and biodegradability may be helpful for fibrocyte regeneration and useful in biomedical applications.	[214,215]
**Gelatin**	Codfish	7.4–24.6 kPa	In situ cross-linked gelatin scaffolds degrade in 7 days	Excellent effect on cell proliferation	Facilitated collagen production and corneal regeneration. Suppressing the inflammatory processes promoting tissue remodeling.	[216,217]
**Chitosan**	Shrimp/crab shell	Porous and mechanically stable	Extended degradation time	Appropriate cytocompatibility and immunomodulatory properties	Bone tissue regeneration and neural tissue regeneration.	[[218], [219], [220]]
**Alginate**	Brown sea algae	Compressive moduli 14.2 kPa	Oxidized alginate hydrogel degraded within 9 days in PBS	competent cell viability	Suitable for cell attachment and spread.	[221]
**Carrageenan**	Red algae	Young modulus values range from 9 to 256 kPa	Only 30 % weight loss on day 21	Significant biocompatibility	Tissue engineering applications.	[222,223]
**Ulvan**	Green seaweeds	37 MPa Tensile strength value 0.58–2.62 N/mm2	Crosslinked ulvan/gelatin scaffolds∼15–20 % weight loss over 96 h	Accelerated fibroblast growth	Enhanced angiogenesis, good for diabetic wound healing.	[56]
**Fucoidan**	Giant squid brown algae	Strong viscoelastic character	Extended degradation time	Promoting cell adhesion and proliferation	Promising for cartilage tissue engineering.	[224,225]

## 7 Safety concerns of marine-derived materials

The safety of marine-derived biomaterials remains a critical concern, as these materials are obtained from different sources containing bioactive molecules with unknown immunogenicity.

Such immune responses can cause inflammation, tissue rejection, and other adverse effects, ultimately compromising the efficacy and safety of tissue repairs. Marine polysaccharides are biocompatible, but some may trigger immune responses depending on their source, extraction method, and purification process. For example, chitosan, derived from shrimp shells, may contain residual proteins like tropomyosin and arginine kinase, which can cause severe inflammation, allergic reactions, and immune rejection [226,227]. The amount of residual protein in chitosan is crucial in determining its suitability for medical applications, with the National Medical Products Administration of China setting the limit for medical-grade chitosan at 0.2 wt%. Similarly, marine proteins like mackerel collagen are known pan-allergens and can lead to hypersensitive reactions if present above a certain threshold [228]. Therefore, the risk of immunogenic responses and potential toxicity must be evaluated before advancing to clinical trials.

Variations in raw material quality, extraction methods, and production techniques can result in batch-to-batch variability, affecting the material’s performance and safety. The extraction process is a common method used to assess the biocompatibility of marine materials by examining the release of substances that may interact with biological systems [229]. ISO standards provide guidance on test sample selection, preparation, and experimental controls. Commercial examples, such as Geltech’s GelMA derived from marine collagen, follow ISO 10993 standards to ensure biocompatibility and safety in tissue engineering applications. Additionally, the identification of marine toxins is crucial for quality control in pharmaceutical development. Recent advancements in analytical methods and molecular biology, including liquid chromatography-mass spectrometry (LC-MS), enzyme-linked immunosorbent assays (ELISAs), and molecular biosensors, enable more sensitive and specific detection of contaminants. These techniques are being refined to increase sensitivity, specificity, and throughput, allowing for more accurate monitoring of toxin levels in marine environments and ensuring the safety of marine-derived products. In line with these advancements, the FDA’s 2024 guidance on biologically sourced materials emphasizes the importance of rigorous testing for contaminants, including marine toxins, to ensure the safety and efficacy of biologically sourced drugs and medical products [230,231]. Consideration of CITES regulations further ensures the legal sourcing of marine-derived materials. Widely used resources such as algal biomass and crustacean shells are not subject to CITES restrictions; however, certain species, including seahorses, are listed in Appendix II of CITES appendices (https://cites.org/eng/app/appendices.php). When CITES-listed organisms are utilized, adherence to permit requirements and traceability measures is essential to guarantee responsible and sustainable use.

Beyond safety, environmental sustainability is another determinant of the clinical and commercial viability of marine biomaterials. Life cycle assessments (LCAs) provide quantitative indicators, such as carbon footprint data, offering insight into the ecological performance of these materials. For example, chitosan production from shrimp-shell waste exemplifies the dual benefit of resource recovery and low carbon emissions, with reported impacts as low as 4.2 g CO_2_-eq/g in optimized Norwegian processes [232,233]. Additionally, alginate production from kelp aquaculture has a carbon footprint ranging from 1.25 to 41.52 kg CO_2_-eq/kg depending on cultivation methods [[234], [235], [236]]. However, quantitative sustainability indicators for marine collagen, particularly carbon footprint data, are limited. A Study by Newton et al. [237] provides life cycle inventories (LCI) for marine ingredients, which can be utilized to estimate the environmental impacts of marine collagen production. This dataset offers insights into the energy consumption, water usage, and greenhouse gas emissions associated with various marine-derived products, including collagen. By applying these LCIs to marine collagen production processes, it is possible to approximate the carbon footprint per kilogram of marine collagen.

These findings emphasize that marine-derived biomaterials contribute not only to tissue regeneration but also to the circular bioeconomy by transforming biological waste into high-value products, reducing environmental impact, and promoting resource efficiency. Integrating safety evaluations with sustainability assessments is therefore essential to advance marine biomaterials toward regulatory approval and clinical translation.

## 8 Conclusions and future prospectives

Marine-derived biomaterials represent a rapidly evolving area of interest in biomedical research, offering a diverse range of naturally sourced compounds with favorable physicochemical and biological characteristics. Their inherent biocompatibility, biodegradability, and bioactivity, combined with sustainable availability, position them as attractive alternatives to conventional synthetic and terrestrial-origin biomaterials. Numerous studies have demonstrated their efficacy in various biomedical fields, including tissue engineering, wound healing, drug delivery, and regenerative medicine. Polysaccharides such as alginate and chitosan, marine collagen, and mineral-based compounds like calcium carbonate have shown significant promise in supporting cellular functions, promoting tissue repair, and enabling targeted therapeutic delivery.

Despite encouraging progress, several challenges must be addressed to fully realize the clinical and commercial potential of marine biomaterials. Variability in raw material quality, limited standardization in extraction and processing techniques, and regulatory complexities remain key barriers to large-scale application. Additionally, a deeper understanding of their interactions with host tissues, long-term biocompatibility, and mechanisms of action is essential for the safe and effective translation of these therapies. Looking ahead, future research should focus on the development of well-characterized, multifunctional marine-derived platforms with tunable properties tailored to specific clinical needs. Advancements in biomaterial engineering, nanotechnology, and biofabrication techniques, such as 3D printing and electrospinning, can further enhance the utility of these materials. Moreover, combining marine-derived compounds with other bioactive agents, stem cells, or exosomes may offer synergistic effects that accelerate healing and regeneration while minimizing adverse immune responses. With continued interdisciplinary collaboration and technological innovation, marine biomaterials hold strong potential to contribute significantly to the next generation of biomedical therapies.

## CRediT authorship contribution statement

**Mohammad Saeed:** Writing – review & editing, Writing – original draft, Data curation, Conceptualization. **Shabnam Anjum:** Writing – review & editing, Writing – original draft, Supervision, Data curation. **Yang Zhang:** Writing – review & editing, Writing – original draft, Supervision, Funding acquisition, Conceptualization.

## Declaration of competing interest

The authors declare that they have no known competing financial interests or personal relationships that could have appeared to influence the work reported in this paper.

## Data Availability

No data was used for the research described in the article.
